# Properties of biopolymer blends based on *Rugulopteryx okamurae* and hydrophobic polycaprolactone (PCL) and hydrophilic acylated soy protein isolated (SPIa)

**DOI:** 10.1007/s11356-024-33659-2

**Published:** 2024-05-15

**Authors:** Ismael Santana, Manuel Felix, Carlos Bengoechea

**Affiliations:** Higher Polytechnic School, University of Seville, Calle Virgen de África, 7, 41011 Sevilla, Spain

**Keywords:** *Rugulopteryx okamurae*, Blend, Seaweed, Soy protein, Functionalization, PCL

## Abstract

The present study explored the utilization of *Rugulopteryx okamurae* (RO), an invasive brown seaweed, as a renewable raw material for plastic materials based on biopolymer blends. The goal of this study was to improve the previously observed poor mechanical properties of materials based on single biopolymer RO. To enhance these properties, two polymers with distinct hydrophobicities were incorporated into the formulation of different blends: hydrophobic polycaprolactone (PCL) and hydrophilic acylated soy protein isolate (SPIa). SPIa was derived from soy protein through a chemical modification process, introducing hydrophilic carboxyl groups. The addition of PCL significantly strengthened the blend, increasing the storage modulus (*E*′_1 Hz_) from ~ 110 to ~ 250 MPa. Conversely, SPIa incorporation resulted in softening, with *E*′ values around 40 MPa. Both additives enhanced deformability proportionally to their concentrations, with SPIa exhibiting notably higher deformability, reaching a maximum deformation of ~ 23% for a RO/SPIa ratio of 25/75. In summary, the study demonstrates the feasibility of producing environmentally friendly blend materials based on RO, tailored for specific applications by incorporating suitable additives into the formulation. Therefore, PCL is recommended for applications susceptible to moisture effects, such as packaging, while SPIa is suggested for highly absorbent applications such as personal care or horticulture.

## Introduction

*R. okamurae* (RO) is an invasive brown seaweed original from northwestern Pacific Ocean (Agatsuma et al. 2005). The great adaptation ability of RO and the similar thermal conditions found in the Mediterranean Sea have promoted the displacement of native seaweed species (García-Gómez et al. 2021). Thus, in 2020, the presence of RO on the seabed of the Strait Natural Park, located on the northern side of the Strait of Gibraltar, ranged from 85 to 96% (García-Gómez et al. 2020). Apart from the damage to ecosystems, significant bioaccumulation occurs on the coasts once the seaweed dies, to the extent that approximately 5000 tons were collected on the beaches of Ceuta between 2015 and 2016 (Ocaña et al. 2016). This situation led to the inclusion of this species of macroalgae in the Spanish Catalogue of Invasive Species in 2020 (García-Gómez et al. 2021). It is also expected to stablish itself in Portugal, Italy, Greece, Croatia, Slovenia, Cyprus and Malta (Altamirano Jeschke and Zanolla 2019).

Seaweed finds various applications in the industry. For instance, its fatty acids can be used for biofuel (Harun et al. 2010), while some pigments are employed in pharmaceuticals and animal feed (Borowitzka 2013). Nonetheless, polysaccharides are the most used component industrially. Some polysaccharides like alginates, agar or fucanes (or fucoidan) are now used on human and animal feed, cosmetics and pharmaceuticals (Berteau and Mulloy 2003; Bixler and Porse 2011; Food and Agriculture Organization of the United Nations 2014). More recently, the development of bioplastics based on seaweed has gained traction due to its ease of processing, enabling the production of biofilms and bio-packaging (Lim et al. 2021; Lomartire et al. 2022).

Poor mechanical properties displayed by some of these seaweed-based plastic materials can be improved when mixed conveniently with another biopolymer. A polymer blend is defined as an intimate mixture of two polymers with no covalent bonds (Asano 2017). Blending permits to produce novel materials with the pursued mixture of properties. No expensive investment is required industrially, as conventional machines can be used for blending to obtain a wide range of properties (Rajeswari et al. 2021). The use of blends based on biopolymers has several advantages when compared to conventional plastics. The first and most obvious is the reduction of non-biodegradable waste and pollution as they are made from biodegradable material. Another advantage is the reduction in the use of pure materials, which can lower production costs by using initially waste material as part of the blend (La Mantia and Morreale 2011).

Polycaprolactone (PCL) is an aliphatic biodegradable and environmental-friendly polyester with a wide range of uses (biomedicine in tissue engineering, drug delivery or food packaging among others Acik et al. 2021; Akay et al. 2022). Its stability, miscibility and hydrophobicity make it a good component for blends, increasing the stress and rigidity of the products (Wróbel-Kwiatkowska et al. 2012; Acik et al. 2021). Moreover, its low glass transition (− 60 °C) and melting temperature (60 °C) ease its processability through injection molding (Malikmammadov et al. 2018).

Soy protein isolate (SPI) is a cheap and available by-product from the soybean oil industry, which have already been demonstrated to be a suitable component of plastic materials (Tian et al. 2012), as SPI bioplastics possess optimal viscoelastic properties which are comparable to those of conventional plastics (Fernández-Espada et al. 2016). The acylation of SPI adds COO^−^ groups that improve the water absorption capacity (Cuadri et al. 2016), which can be interesting in the production of green superabsorbent materials, with applications in the diapers or personal care sector.

Both SPI and PCL are biodegradable polymers which potential in the formulation of blends containing seaweed materials was considered in this study. These polymers have already been processed through injection molding to produce bioplastics, such as the one proposed by Cuadri et al. (Cuadri et al. 2017) for functionalized soy protein, or the crayfish protein-PCL biocomposites produced by Felix (Félix et al. 2015). Even if blends have been obtained by using natural biopolymers (e.g. albumen, soy) (Martín-Alfonso et al. 2014), biodegradable synthetic polymer (e.g. PCL) (Félix et al. 2015) or even inorganic particles (e.g. Montmorillonite) (Felix et al. 2018), to the best of our knowledge, there are not results of blends based on seaweed materials together with either by SPI or by PCL.

The aim of this work was to obtain different blends using RO as matrix and including either acylated SPI (SPIa) or PCL. The active matter/glycerol ratio was kept constant at 50/50, assessing the effect of different RO/PCL and RO/SPI ratios (RO/PCL ratios: 100/0, 90/10, 80/20 and 70/30; RO/SPIa ratios: 100/0, 75/25, 50/50 and 25/75). Subsequently, blends were processed by injection molding at 120°C, and they were characterised by DMA, tensile tests, water absorption capacity and scanning electron microscope (SEM) microscopy.

## Materials and methods

### Materials

The *R. okamurae* (RO) seaweed used to produce the composites was live seaweed collected from the seabed of the Bay of Algeciras and subsequently freeze-dried. It was supplied by the Andalusian Institute for Agricultural, Fisheries and Organic Production Research and Training (IFAPA, Puerto Real, Spain). The freeze-dried seaweed was grounded on a kitchen blender (Mambo10070, CECOTEC, Valencia, Spain) at maximum speed, obtaining a particle size diameter D [4,3] within the range of 10 to 100 µm for most particles (~ 90%).

Polycaprolactone (PCL) was used as part of the blend with an average molecular weight of 80 kDa (Sigma-Aldrich, San Luis, MO, USA). Soy protein isolate (SPI) (SUPRO 500E IP, Leper, Belgium) was supplied by PROANDA (Proveedora Andaluza, S.L., Sevilla, Spain). The acylation of SPI was carried out according to a modification of the method proposed by Hwang et al. (Hwang and Damodaran 1996a, b) and modified by Cuadri et al. (2016). Succinic acid (SA) was incorporated into a 4 wt% soy protein isolate (SPI) solution in distilled water to achieve a SA/SPI ratio of 0.12. The solution was then stirred for 1 h while ensuring that the pH remained between 7.5 and 8.5 by titrating with a 3.0 N NaOH solution as needed. After 1 h, 3.0 N HCl was added to adjust the pH to 7 and halt the reaction. Subsequently, the SPI solution was dialyzed against distilled water for 3 days, with periodic water changes to eliminate any residual salts from SA. Finally, the acylated SPIs were freeze-dried using a Teslar LyoQuest (Teslar, Life Science Solutions, Madrid, Spain) to obtain the final SPIa powder. Glycerol (Gly) (Panreac Química, S.A., Castellar del Vallès, Spain), was employed as the plasticizer.

### Methods

#### Sample preparation

A two-step processing method has been followed to obtain the final conveniently shaped blends: (i) active matter (RO, PCL, SPIa) and plasticizer (Gly) were mixed in a two-blade counter-rotating batch mixer Haake Polylab QC (ThermoHaake, Karlsruhe, Germany) at 50 rpm and 60 °C for PCL blends or room temperature for SPIa composites until homogeneity (5 min). Blends at 90/10, 80/20 and 70/30 RO/PCL ratios as well as 75/25, 50/50 and 25/75 RO/SPIa ratios were always obtained keeping a 50/50 biomass/Gly ratio, as PCL does not require plasticizer to be processed; (ii) blends were injected in a Minijet II (ThermoHaake, Karlsruhe, Germany) using same conditions proposed by Álvarez-Castillo et al. (2019). The processing conditions were cylinder temperature of 60°C, a mold temperature of 120 °C, injection pressure of 500 bar (10 s) and holding pressure of 200 bar (140 s), obtaining 1 × 10 × 60 mm^3^ probes.

#### Characterization of blends

##### Rheological characterization

Blends obtained after the mixing stage were first analysed by dynamic mechanical thermal analysis (DMTA) using a DMA850 (TA Instruments, Wakefield, MA, USA) in compression mode. Frequency tests, from 0.1 to 10 Hz at a constant temperature of 25 °C, and temperature ramp tests, from 0 to 180 °C at a constant frequency of 1 Hz, were performed. Both tests were carried out within the linear viscoelastic region, previously determined by strain sweep tests at 1 Hz. Fifteen-millimetre diameter parallel plates geometry was used for DMTA tests.

Rectangular probes (60 mm × 10 mm × 1 mm) of injection molded blends were also analysed in the DMA850 (TA Instruments, Wakefield, MA, USA), but using a tension clamp geometry. Frequency sweep tests, from 0.1 to 10 Hz, at a constant temperature of 25 °C, and temperature ramp tests, from 0 to 180 °C, at a constant frequency of 1 Hz, were performed within the linear viscoelastic region, previously determined by strain sweep tests at 1 Hz.

##### Tensile properties

Uniaxial tensile tests were performed using rectangular probes (60 mm × 10 mm × 1 mm) of injection molded blends according to the standard ISO 527–2 (ISO 2012). A constant elongation rate of 1 mm·min^−1^ and room temperature in a RSA3 (TA Instruments, MA, USA) were employed to obtain stress–strain curves. From these curves, three mechanical properties were determined: Young’s modulus (*E*), maximum stress (σ_max_) and maximum strain (ε_max_).

##### Water Uptake Capacity

Water uptake capacity (WUC) of injection molded blends was determined following a three-stage method described by Cuadri et al. (2018). First, blends were dried at 50 °C for 24 h and weighed (*w*_1_). After that, dried samples were immersed in 100 mL of deionized water for 24 h and weighed (*w*_2_). Finally, samples were lyophilized for 24 h and weighed (*w*_3_). WUC and soluble matter loss (SML) can be estimated using Eqs. 1 and 2.

1 $$WUC \;\left(\%\right)=\frac{\left({w}_{2}-{w}_{3}\right)}{{w}_{3}}.100$$

2 $$SML \;\left(\%\right)=\frac{{(w}_{1}-{w}_{3})}{{w}_{1}}.100$$

##### Scanning electron microscopy

After WUC, freeze-dried samples obtained after water uptake tests were cut into small pieces (~ 2.5 mm), gold coated and examined by SEM with a ZEISS EVO microscope (Oberkochen, Germany), employing a beam current of 18 pA at a working distance of ~ 8 mm. The acceleration voltage was of 10 kV. Images obtained were analysed by ImageJ (Bethesda, MD, USA).

### Statistical analysis

STATGRAPHICS Centurion XVIII software (The Plains, VA, USA) was used to carry out the statistical analysis by ANOVA tests. All measurements were carried out at least in triplicate and significant difference (*p* < 0.05) was reported in selected parameters by superscript letters.

## Results and discussion

### Rheological properties of blends during mixing

**Fig. 1:**
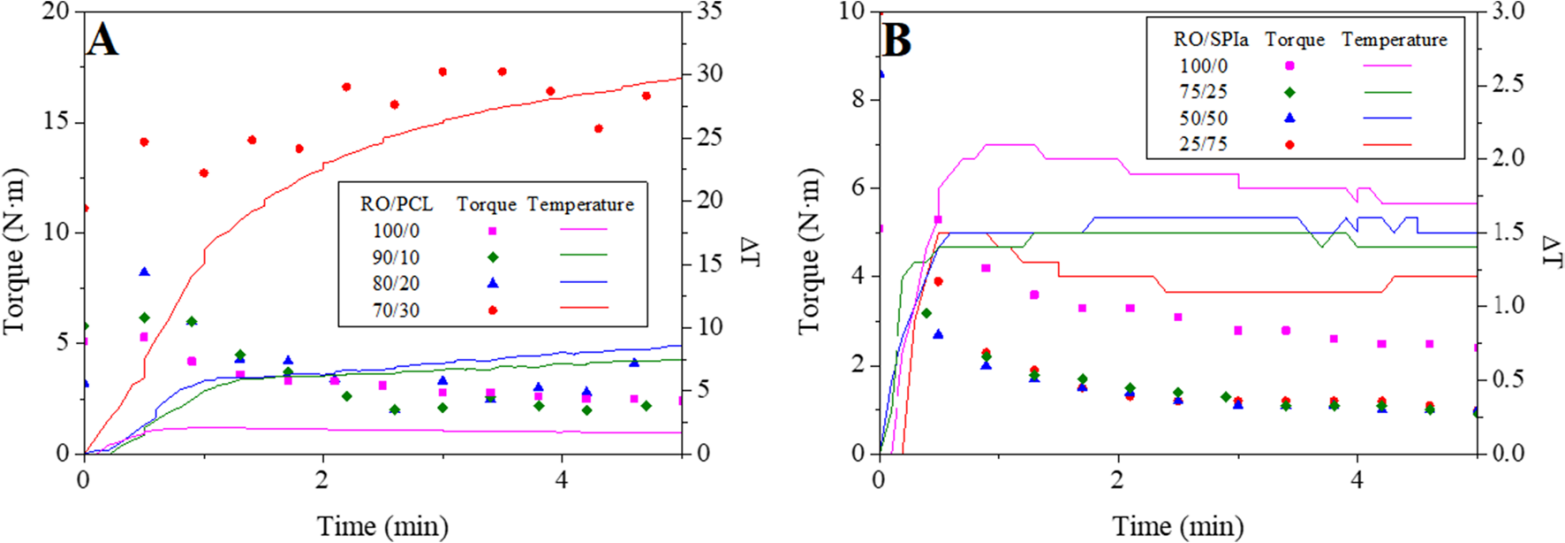
Torque and Δ*T* (where *T* is the temperature over mixing and *T*_0_ is ~ 60 °C for (**A**) and room temperature for (**B)**) for (**A)** different RO-PCL/Gly and (**B**) different RO-SPIa/Gly systems during the mixing stage

Figure 1 depicts the torque and temperature evolution over mixing time for the studied blends containing different ratios of RO/PCL (100/0, 90/10, 80/20 and 70/30) (Fig. 1A) or RO/SPIa (100/0, 75/25, 50/50 and 25/75) (Fig. 1B). Generally, a peak torque value is observed in the initial mixing stage, followed by a decrease until a plateau zone is reached. This trend is consistent regardless of the additive used, except for the system with a RO/PCL ratio of 70/30, where the torque continues to increase until reaching a plateau region, without observing a distinct peak value. This behavior resembles that found by Felix et al. (2016) for SPI plasticized with glycerol and sorbitol, where sorbitol was added as a solid, suggesting that the 70/30 RO/PCL blend may exhibit a predominant solid behavior. It is noteworthy that increasing the percentage of PCL in the blend (Fig. 1A) results in lower plasticizer content in the mixture, leading to an increase in torque and temperature. Reduction in plasticizer content reduces the free volume in the blend, resulting in greater friction between polymer chains and consequently higher temperature increase (Jerez et al. 2005). Conversely, when SPIa is used as an additive in the blends (Fig. 1B), a decrease in torque and a lower temperature rise (Δ*T*) are observed, with torque for all RO/SPIa composite systems eventually stabilizing around 1 N·m. Unlike PCL, it appears that SPIa promotes the fluid behavior of these samples. Δ*T* initially increases during the mixing process, eventually reaching a constant value when the torque plateaus. Similar trends were observed for RO/Gly systems not containing SPIa, where higher seaweed content in the mixture resulted in higher torque and temperature values (Santana et al. 2022). Therefore, the highest Δ*T* (30 °C) is observed for the 70/30 RO/PCL system due to the higher torque values associated with lower glycerol content, leading to greater mechanical energy dissipation (Pommet et al. 2003). Notwithstanding, it should be noticed that the mixing process of blends containing PCL started at 60 °C since that is the melting point of PCL, as previously practiced in works where PCL was processed with other biopolymers (Félix et al. 2015; Thongpin et al. 2020).

### Dynamic mechanical thermal analysis

#### Blends after mixing

**Fig. 2:**
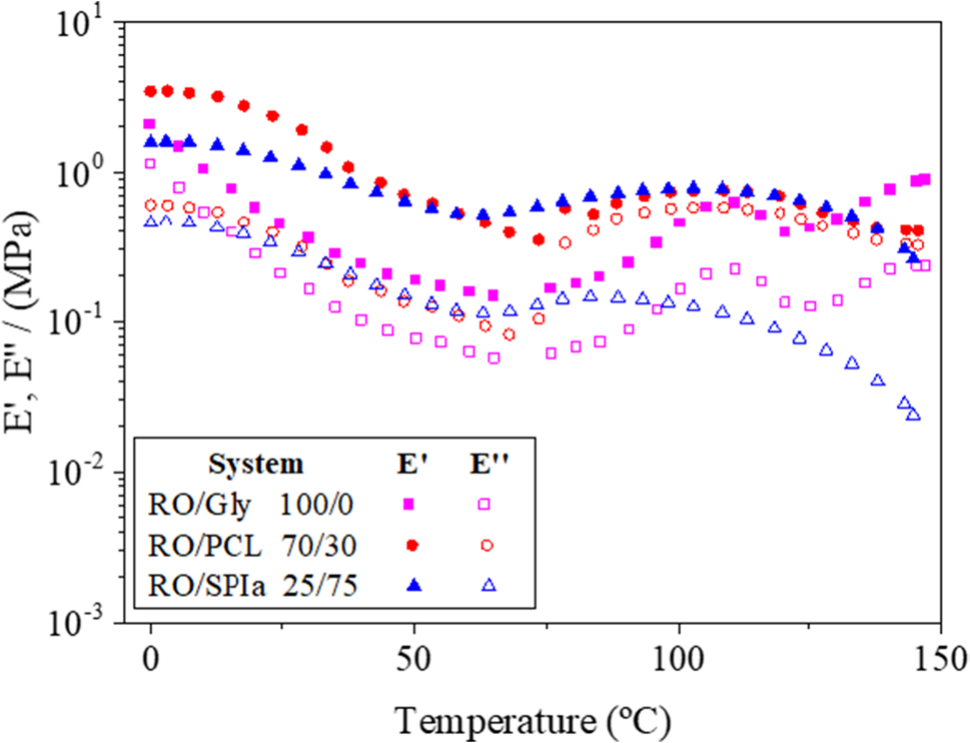
Evolution of *E*′ and *E*″ with temperature for the reference system (RO/Gly, 50/50) and the two limit conditions: systems 70/30 (RO/PCL) and 25/75 (RO/SPIa)

Figure 2 shows the evolution of viscoelastic moduli (*E*′ and *E*″) with temperature (from 0 to 150 °C) for the reference system containing only the plasticizer as additive (RO/Gly) and selected blends (RO/PCL 70/30 and RO/SPIa 25/75) obtained after the mixing stage.

A predominantly elastic behavior is observed across the entire temperature range studied, and the addition of polymers (PCL and SPIa) as additives enhances the elastic modulus of the materials, thereby improving their mechanical properties. Throughout the temperature range, *E*′ remains consistently higher than *E*″, even after PCL is melted (i.e. above 65 °C). This indicates that RO prevents complete melting of the RO/PCL material, while PCL enhances the viscoelastic properties of the blend.

However, all the systems evaluated exhibited a certain dependence of the viscoelastic moduli with temperature. In the case of RO/SPIa systems, there is a decrease in the elastic modulus until a minimum is reached at 65–70 °C. This minimum likely corresponds to the glass transition of SPIa, consistent with results obtained by Cuadri et al. (2016) for SPIa/Gly hydrogels. Materials containing only RO as a polymer demonstrate thermosetting potential at high temperatures (i.e. above 75 °C), confirming the lack of interactions during the mixing stage and its ability to reinforce the microstructure through processing (Jerez et al. 2007; Santana et al. 2022). Similar findings have been reported for other systems, such as crayfish or albumen, which exhibit properties suitable for further processing (Romero et al. 2009; Felix et al. 2017).

#### Injection molded blends

**Fig. 3:**
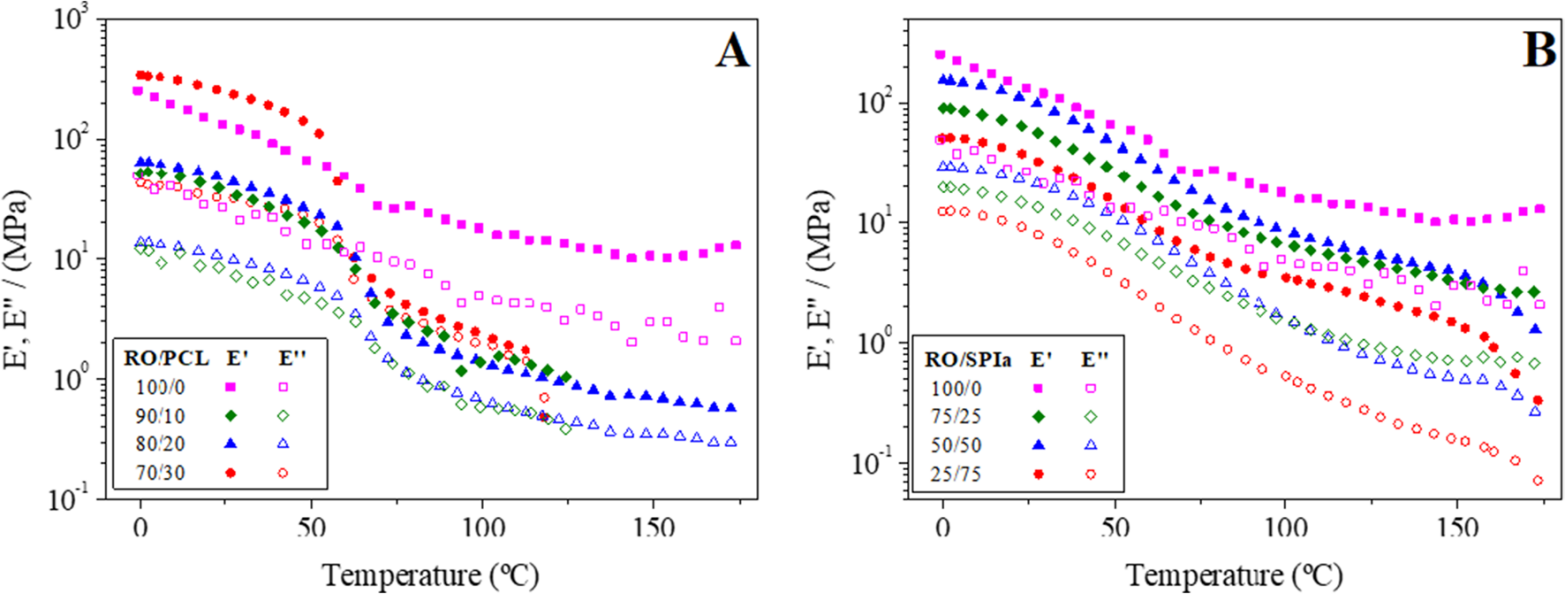
Evolution of elastic (*E*′) and viscous (*E*″) moduli with temperature for (**A**) RO/PCL blends at 100/0, 90/10, 80/20 and 70/30 ratios, and (**B**) RO/SPIa blends at 100/0, 75/25, 50/50 and 25/75 ratios

Figure 3 illustrates the dynamic mechanical thermal analysis (DMTA) tests conducted from 0 to 180 °C for the blends comprising RO/PCL at four ratios (100/0, 90/10, 80/20 and 70/30) (Fig. 3A) and RO/SPIa at four ratios (100/0, 75/25, 50/50 and 25/75) (Fig. 3B). A general decrease in the value of the elastic modulus (*E*′) is observed for both blends with increasing temperature, indicating a softening of the materials attributed to the disruption of secondary interactions (e.g. hydrogen bonds). The continuous decrease in *E*′ throughout the DMTA tests for all samples suggests that there is no potential for thermosetting to be achieved with further increases in molding temperature. However, the reference system containing only RO biomass exhibits a slight increase in the viscoelastic moduli at higher temperatures.

Furthermore, the significant increase in the elastic modulus values from blends obtained right after mixing to those injection molded by approximately one or two orders of magnitude, depending on the RO/filler ratio, suggests that the temperature and pressure conditions during the injection molding stage were effective in achieving substantial strengthening of the materials beyond mere mixing. This outcome also affirms the suitability of the processing technique employed in producing these materials.

An inflection point is observed at 60 °C in Fig. 3A, corresponding to the melting point of PCL (McKeen 2021), which is not evident in Fig. 3B. Higher PCL content results in a higher elastic modulus (*E*′) at lower temperatures. Additionally, a slight inflection point is observed for the RO/SPIa systems around 55–70 °C, possibly corresponding to a glass transition of the sample. This thermal event has been previously documented by Fernández-Espada et al. (2016) for SPI-based bioplastics and by Cuadri et al. (2017) for functionalized soy protein bioplastics (SPIa). It is noteworthy that the application of these blends becomes more restricted when PCL is added due to its thermal instability beyond its melting point (60 °C), often resulting in the fracture of injection molded blends before reaching 180 °C in most cases. This has been observed previously by Félix et al. (2015) for crayfish-PCL blends. However, SPIa composites demonstrate higher resistance to elevated temperatures.

An inflection point can be observed at 60 °C in Fig. 3A, which is not observed in Fig. 3B, and corresponds with the melting point of PCL (McKeen 2021). In this case, higher content on PCL resulted in higher elastic modulus (*E*′) at lower temperatures. Moreover, there is a slight inflection point for the RO/SPIa systems around 55–70 °C, which may correspond to a glass transition of the sample. This thermal event was already observed by Fernández-Espada et al. (2016) for SPI-based bioplastics, and by Cuadri et al. (2017) for functionalized soy protein bioplastics (SPIa). Please notice that the application of these blends is more restricted when adding PCL due to its thermal instability beyond its melting point (60 °C), resulting in a breaking of blends before 180 °C in most cases, as already observed by Félix et al. (2015) for crayfish-PCL blends. However, SPIa composites demonstrate higher resistance to elevated temperatures.

**Fig. 4:**
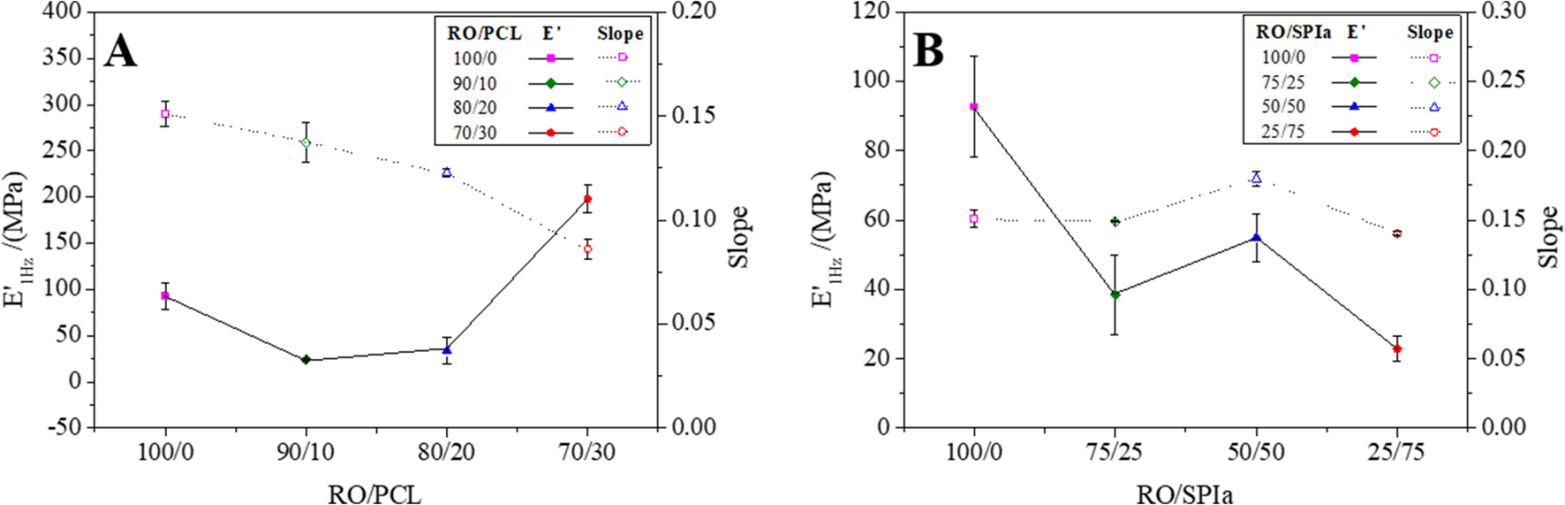
Evolution of elastic (*E*′) modulus at 1 Hz and the slope of frequency after heat treatment for (**A**) RO/PCL blends at 100/0, 90/10, 80/20 and 70/30, and (**B**) RO/SPIa blends at 100/0, 75/25, 50/50 and 25/75

Figure 4 shows the *E*′ at 1 Hz (*E*′_1_) and the slope of *E*′ versus frequency of the injection molded blends as a function of the PCL (Fig. 4A) and SPIa (Fig. 4B) content. Figure 4A shows an increasing trend in the value of the *E*′_1 Hz_ in the RO/PCL systems when the amount of PCL increases. This result may involve a greater interaction between the polymeric chains since plasticizer decreases as the PCL ratio increases. This solid-like behavior of PCL on blends produces a more rigid structure. Furthermore, these lower interactions agree with the decrease in the slope when the amount of PCL in the mixture increases, which implies a lower dependence between the elastic modulus and the frequency under these conditions (i.e. higher relaxation time) (Ferry 1980).

On the other hand, when the dependence of the *E*′_1 Hz_ value on the RO/SPIa ratios is analysed (Fig. 4B), a general tendency onto lower values is observed as SPIa content is increased. When the RO/SPIa ratio percentage goes from 100/0 to 25/75, a decrease of interactions between the biopolymers is deduced. As the presence of SPIa promotes a fluid-like behaviour, it produces a more deformable structure and, therefore, less rigid. In this case, the slopes do not vary greatly with the SPIa content, especially when compared to RO/PCL systems (Fig. 4A), where a continuous decrease is detected, which may be related to the relatively low melting point of PCL.

### Tensile tests

**Fig. 5:**
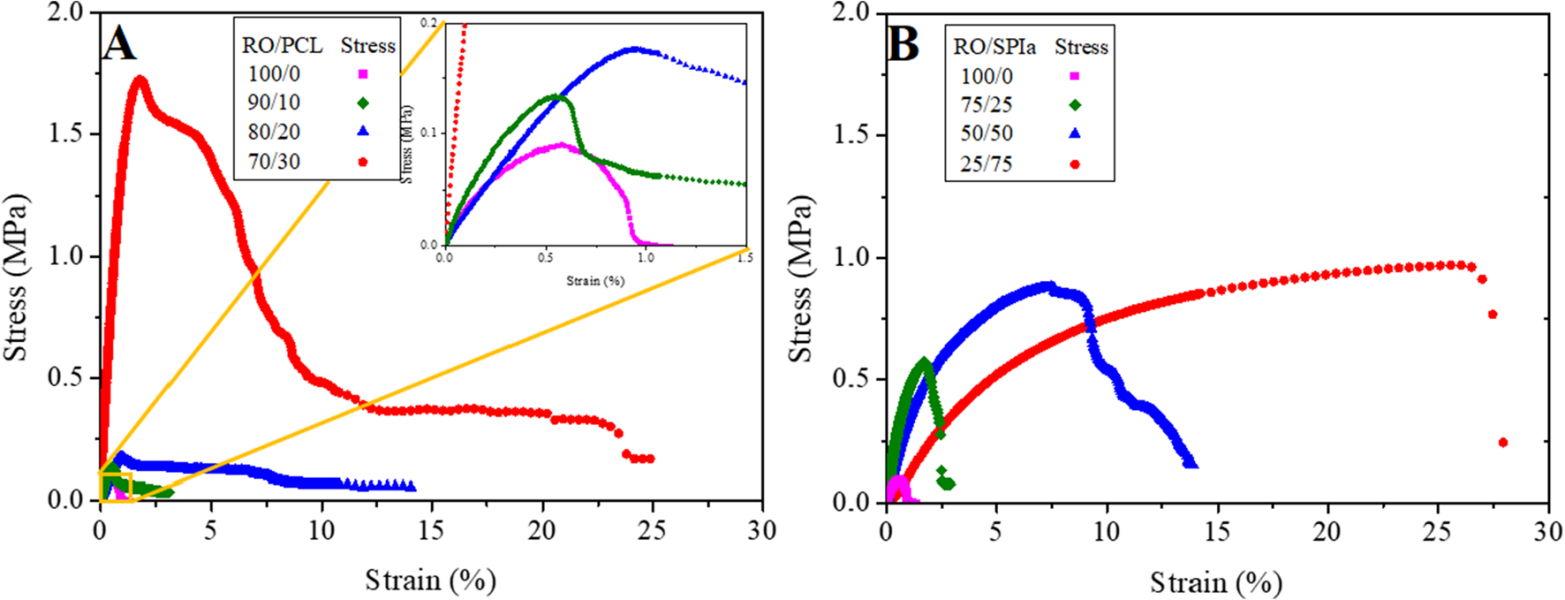
Stress–strain curves for blends with (**A**) different RO/PCL ratios (100/0, 90/10, 80/20 and 70/30) and (**B**) different RO/SPIa ratios (100/0, 75/25, 50/50 and 25/75)

Figure 5 shows the stress–strain curves obtained by the RO/PCL blends (100/0, 90/10, 80/20 and 70/30) (A) and the RO/SPI blends (100/0, 75/25, 50/50 and 25/75) (B) studied. All systems show an initial linear elastic region from which Young’s modulus, *E*, can be determined at relatively low strain values where exclusively linear elastic deformation takes place. As strain increases, the slope begins to decrease slightly, indicating the onset of plastic deformation in the material. This continues until a maximum stress value (σ_max_) is reached. Upon surpassing this threshold, there is an abrupt decline in slope, corresponding to the fracture of the probe at a maximum strain (ε_max_).

The fracture behaviour of probes appears to vary depending on the additive incorporated into the blends (PCL or SPIa). In RO/PCL systems, a peak stress is followed by prolonged deformation before eventual rupture, whereas in RO/SPIa systems, rupture occurs more abruptly once the maximum stress (σ_max_) is surpassed. In PCL composites, the probe exhibits localized deformation when maximum stress is reached, a phenomenon not observed in SPIa blends. The higher concentration of plasticizer in RO/SPIa systems compared to RO/PCL systems results in a transition from plastic to more elastic fracture behavior at higher deformations, consistent with findings by Chang et al. (2006).

Table 1 shows the mechanical parameter values (*E*, ε_max_ and σ_max_) for the reference (50/50, RO/Gly), RO/PCL blends (90/10, 80/20 and 70/30) and RO/SPIa blends (75/25, 50/50 and 25/75). Higher values of ε_max_ and σ_max_ are obtained with increased amounts of PCL or SPIa in the formulation. Notably, an important increase in σ_max_ is observed at the highest PCL ratio, while in RO/SPIa blends, the most significant difference is observed in the ε_max_ value, increasing by an order of magnitude at 75% SPIa (up to 22.8 ± 3.51). This heightened deformability is primarily associated with the plasticizer. PCL, as a biopolymer, enhances the mechanical properties (*E*, σ_max_ and ε_max_) as its content increases, bringing the blends closer to the attributes of this biodegradable synthetic polymer, aligning with values typically obtained for PCL (La Mantia and Morreale 2011).

**Table 1:** Mechanical parameters (Young’s modulus (*E*), maximum stress (σ_max_) and maximum strain (ε_max_)) for blends with different (A) RO/PCL ratios (70/30, 80/20, 90/10 and 100/0) and (B) RO/SPIa ratios (25/75, 50/50, 75/25 and 100/0)

	System	E(MPa)	ε_max_ (%)	σ_max_ (MPa)
Reference	**100/0**	0.40 ± 0.14^a^	0.49 ± 0.07^a^	0.088 ± 0.021^a^
RO/PCL	**90/10**	0.47 ± 0.046^a^	0.64 ± 0.14^a^	0.12 ± 0.031^a^
	**80/20**	0.28 ± 0.068^a^	1.01 ± 0.14^b^	0.17 ± 0.012^b^
	**70/30**	1.64 ± 0.48^b^	2.24 ± 0.66^c^	1.70 ± 0.19^c^
RO/SPIa	**75/25**	0.54 ± 0.16^a^	2.21 ± 0.55^c^	0.50 ± 0.086^d^
	**50/50**	0.44 ± 0.064^a^	9.06 ± 1.13^d^	0.91 ± 0.084^e^
	**25/75**	0.093 ± 0.058^c^	22.8 ± 3.51^e^	0.78 ± 0.15^e^

Different letters within a column indicate significant differences (*p* < 0.05)

In contrast, when SPIa is utilized to form RO/SPIa blend materials, an increase in deformation at break is observed. This behavior could be attributed to the hydrophilic nature of SPIa, which interacts more effectively with the plasticizer (Gly) compared to PCL, as both are highly hydrophilic (Sanyang et al. 2015). Regarding Young’s modulus values (*E*), no significant differences are observed between 100/0, 90/10 and 80/20 RO/PCL systems, and 100/0, 75/25 and 50/50 RO/SPIa systems. However, a significant increase is noted at the highest percentages, i.e. 70/30 RO/PCL and 25/75 RO/SPIa systems, consistent with results obtained from DMTA tests. Similar findings were reported by Félix  et al. (2015) for crayfish/PCL blends and by Fernández-Espadas et al. (2016) for SPI/Gly bioplastics.

### Water uptake capacity

**Fig. 6:**
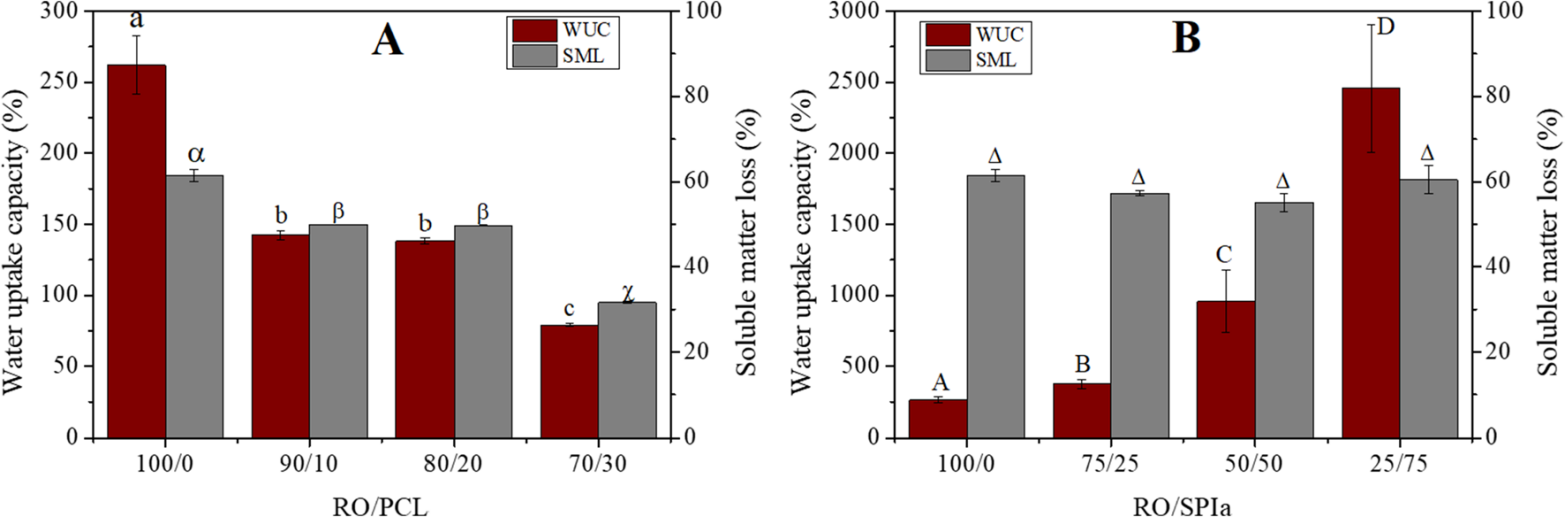
Water uptake capacity and soluble matter loss for (**A**) RO/PCL systems at 100/0, 90/10, 80/20 and 70/30 ratios and (**B**) RO/SPIa systems at 100/0, 75/25, 50/50 and 25/75 ratios. Different letters above the same parameter (WUC or SML) indicates significant differences (*p* < 0.05). Letters used to denote significant differences in both figures are independent of each other

Figure 6 presents the water uptake capacity (WUC) and soluble matter loss (SML) of RO/PCL (100/0, 90/10, 80/20 and 70/30) (A) and RO/SPIa (100/0, 75/25, 50/50 and 25/75) (B) injection molded blends. In the case of PCL blends, a decrease in the amount of PCL results in an increase in WUC (262.15 ± 20.60% for RO/PCL 100/0 and 78.89 ± 1.18% for RO/PCL 70/30). This trend suggests that blends with higher RO content also exhibit higher glycerol (Gly) content, promoting the formation of pores in the structure and, thus, increasing absorption sites, as Gly tends to migrate to the aqueous phase due to its hydrophilic behavior (Félix et al. 2014). The formation of interconnected pores is crucial for enhancing water uptake capacity (Guillard et al. 2013; Felix et al. 2018).

Consequently, the diffusion of water molecules is hindered when PCL is present in the material structure, leading to a reduction in final water adsorption. The hydrophobic nature of PCD impedes water diffusion through the polymeric matrix (Arakawa and DeForest 2017). Conversely, an increase in SPIa content (Fig. 6B) results in higher water uptake capacity, attributed to the high hydrophilicity of SPIa (Cuadri et al. 2016). Notably, the 25/75 ratio (RO/SPIa) exhibits a WUC exceeding 1000% (specifically, 2455.99 ± 449.74%), classifying it as a superabsorbent material (SAM), capable of absorbing from 10 to 100 times its own weight in water or fluids (Buchholz and Graham 1998). The pronounced hydrophilicity of SPIa in the blend is attributed to the acylation process applied as a pretreatment to SPI, which enhances water absorption compared to untreated SPI (Cuadri et al. 2016). Previous studies have also demonstrated the production of superabsorbent materials using SPIa (Cuadri et al. 2016). However, the proposed materials achieve comparable values using a lower quantity of soy protein, thus holding greater industrial interest.

Regarding SML, an increase in its value is observed with higher Gly content in the RO/PCL formulation (which corresponds to a decrease in PCL content). This increase may be attributed to the solubilization of glycerol, known for its pronounced hydrophilic behavior, although biomass loss also occurs alongside glycerol loss, resulting in a mass loss exceeding the percentage of glycerol in the sample, akin to observations in bioplastics based solely on seaweed. Conversely, in RO/SPIa systems, Gly content remains constant across different systems, with no significant differences observed in SML values.

### Scanning electron microscopy

**Fig. 7:**
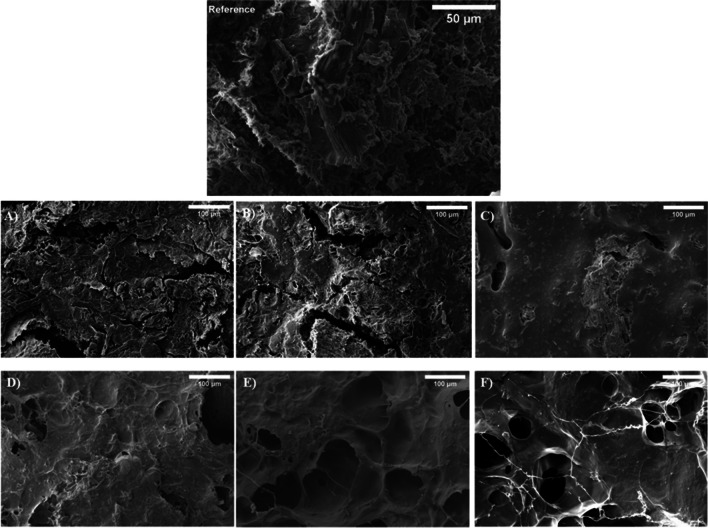
Scanning electron microscopy images RO/GLY 50/50 (without PCL or SPIa) as reference, RO/PCL 90/10 (**A**), 80/20 (**B**) and 70/30 (**C**) systems; and RO/SPIa 75/25 (**D**), 50/50 (**E**) and 25/75 (**F**) systems after 24 h of water immersion and lyophilized with a magnification of 500 × for reference and 200 × for the blends

Figure 7 depicts the SEM images of all freeze-dried blend systems after water immersion. Figure 7A, B and C correspond to RO/PCL systems with ratios of 90/10, 80/20 and 70/30, respectively. Figures 7D, E and F represent systems with RO/SPIa ratios of 75/25, 50/50 and 25/75, respectively.

The SEM image in Fig. 7C illustrates that with an increase in PCL content in the RO/PCL systems, the surface of the blends becomes smoother, indicating a reduction in the number of pores in the 70/30 RO/PCL system. This suggests fewer water absorption sites and interconnections. Conversely, lower PCL contents (e.g. RO/PCL 80/20 and 90/10 systems) exhibit greater roughness and cracks (Figs. 7A, B). These systems show minimal differences among them, consistent with their similar and higher water uptake capacities compared to the 70/30 RO/PCL system (Fig. 7C). Additionally, the RO/PCL 70/30 system demonstrates a higher viscoelastic modulus, indicating a more resilient and rigid structure, as observed in Fig. 5A, while the 80/20 and 90/10 systems exhibit negligible differences between them. These findings align with those of Zein/PCL blends, which exhibited a porous and cracked structure, with reduced water absorption at higher PCL contents (Wu et al. 2012).

In contrast, the SEM images of RO/SPIa systems in Fig. 7 reveal more pronounced differences with changes in their ratios. Specifically, pore size increases with higher SPIa percentages in the mixture, along with an increase in water uptake capacity. Image F, corresponding to the RO/SPIa 25/75 system, exhibits numerous imperfections and larger pores compared to images E (RO/SPIa 50/50) and D (RO/SPIa 75/25). These outcomes are consistent with those reported for bioplastics derived from acylated soy protein isolate (Cuadri et al. 2017). Comparison of these results with those obtained from dynamic mechanical thermal analysis (DMTA) reveals that the RO/SPIa 25/75 system has the lowest viscoelastic moduli (Fig. 3B) and the least rigid structure observed in tensile tests (Fig. 5B). This confirms a more flexible structure with a greater swelling ability when immersed in water.

## Conclusions

The utilization of *R. okamurae* seaweed in blend production facilitates the production of materials through injection molding, employing a hydrophobic additive (polycaprolactone) and a hydrophilic additive (acylated soy protein isolate) as tuning agents, alongside glycerol as a plasticizer. Increasing the proportion of polycaprolactone in the blends led to blends exhibiting elevated values of viscoelastic moduli, rigidity and resistance, achieving maximum deformation (2.24 ± 0.66%) and stress (1.70 ± 0.19 MPa) at a 70/30 RO/PCL ratio. Conversely, elevating the proportion of acylated soy protein isolate in the RO/SPIa systems resulted in blends with reduced viscoelastic moduli, yielding more flexible and deformable structures, with deformations ranging from 2.21 ± 0.55% to 22.8 ± 3.51% as the RO/SPIa ratio increased from 75/25 to 25/75, respectively.

Dynamic mechanical analysis (DMA) tests indicated softening upon heating for both blends, albeit with prevailing elastic behavior. Water uptake capacity (WUC) tests confirmed DMA and tensile test results. With RO/PCL, increasing the amount of PCL yielded a stiffer structure with some pores resulting from water absorption, as observed in SEM images. Conversely, RO/SPI systems exhibited increased porosity and WUC with higher SPIa content, reaching values of 2455 ± 449 wt% at a 25/75 RO/SPIa ratio, indicating a superabsorbent material.

These findings illustrate the versatility of different formulations in blend production, allowing modulation of material properties based on *R. okamurae* seaweed. Blending with PCL yields hydrophobic and durable materials, while the presence of SPIa produces hydrophilic and malleable materials. In conclusion, this study contributes to sustainable materials development, aligning with the trend of eco-friendly blends. Through comprehensive analysis, it offers insights into seaweed-based blends, providing potential solutions to environmental challenges and exploring novel applications in materials science.

## Data Availability

The data required to reproduce these findings cannot be shared at this time as the data also forms part of an ongoing study.
